# Don't spin the pen: two alternative methods for second-stage sampling in urban cluster surveys

**DOI:** 10.1186/1742-7622-4-8

**Published:** 2007-06-01

**Authors:** Rebecca F Grais, Angela MC Rose, Jean-Paul Guthmann

**Affiliations:** 1 Epicentre, 8, rue Saint Sabin, 75011 Paris, France

## Abstract

In two-stage cluster surveys, the traditional method used in second-stage sampling (in which the first household in a cluster is selected) is time-consuming and may result in biased estimates of the indicator of interest. Firstly, a random direction from the center of the cluster is selected, usually by spinning a pen. The houses along that direction are then counted out to the boundary of the cluster, and one is then selected at random to be the first household surveyed. This process favors households towards the center of the cluster, but it could easily be improved. During a recent meningitis vaccination coverage survey in Maradi, Niger, we compared this method of first household selection to two alternatives in urban zones: 1) using a superimposed grid on the map of the cluster area and randomly selecting an intersection; and 2) drawing the perimeter of the cluster area using a Global Positioning System (GPS) and randomly selecting one point within the perimeter. Although we only compared a limited number of clusters using each method, we found the sampling grid method to be the fastest and easiest for field survey teams, although it does require a map of the area. Selecting a random GPS point was also found to be a good method, once adequate training can be provided. Spinning the pen and counting households to the boundary was the most complicated and time-consuming. The two methods tested here represent simpler, quicker and potentially more robust alternatives to spinning the pen for cluster surveys in urban areas. However, in rural areas, these alternatives would favor initial household selection from lower density (or even potentially empty) areas. Bearing in mind these limitations, as well as available resources and feasibility, investigators should choose the most appropriate method for their particular survey context.

## Background

Over time, the World Health Organization Expanded Program on Immunization (EPI) cluster survey design has become the default choice in the field to measure vaccination coverage and other indicators, even when a sampling frame is available. The cluster method was developed in the 1970s for immunization coverage in the USA and expanded for the smallpox eradication campaign later that decade [1,2]. In a two-stage cluster design, the first stage involves selection of clusters throughout the survey area; this is usually done proportionally to estimated population size. The next stage concerns selection of the first sample household within the cluster. The method has been described in detail in previous publications and manuals [3-8].

Despite criticism and recommendations for its modification, [9-13] these are rarely taken into consideration and the 'original' methodology may be followed by default. In this paper, we focus on one problematic aspect of the cluster survey as it is often implemented: selection of households in the second stage. Although the original method called for a list of households to be selected at random from a list of all eligible households, this is not usually implemented, as such lists are rarely available, especially in settings where cluster surveys are often performed. Instead, variations on a "random walk" are widely used. In perhaps the most common implementation, survey teams select a random starting direction from a central location in the cluster by spinning a pen or bottle. Households lying on this transect from the center to the border of the cluster are counted and one of them is then chosen at random. Proximity selection is then used to select subsequent households as the "next nearest" until the desired sample size is reached.

"Spinning the pen" is often justified as a way to avoid costly and time-consuming listing of all households in the selected cluster in the absence of a sampling frame. As long as the starting point is selected randomly and probabilities of selection can be calculated (the number of households selected divided by the total number eligible for selection from the sampling frame), thus obtaining a probability sample, supporters claim the method can be considered unbiased [14]. However, these two conditions are rarely met. Firstly, by starting household selection from the center of the cluster, households near the center are more likely to be selected than outlying households. As described by Brogan et al, [9] if we assume the sampling area or cluster is any randomly selected rectangle extending from the presumed center to its boundary, and if all possible rectangles are drawn, households in the center will fall into most rectangles, while households on the periphery will not. Secondly, although probabilities of selection can theoretically be calculated, given that the total number of households in the cluster are known, this is only very rarely the case, [11,12,15] as the number of eligible households in an area is not always available. Thus, the EPI method cannot reflect true probability sampling, both due to its bias of selecting households towards the center of the area being sampled (so that these households have a greater probability of selection than those on the periphery) and because the actual probability of any household being selected is rarely known, or calculated. An additional problem is that the spin-the-pen method relies on interviewer judgment to identify the random starting direction.

As part of a larger survey conducted by Epicentre to measure meningitis vaccination coverage after a mass campaign in the town of Maradi, Niger, we implemented and then compared the spin-the-pen method to two alternative methods for selection of the first household to be surveyed: selection of a random point using a Global Positioning System (GPS), and use of a sampling grid. These did not entail significant methodological deviations from the survey protocol, required little training, and we believed that they could be conducted quickly. Variations on both of these alternative methods have been used in previous retrospective cluster surveys [16,17] and have been suggested as potential alternatives [18], but have not been compared in the field to the spin-the-pen method. Here, we discuss the implementation of these methods in an informal study that compared the spin-the-pen method with these two simple alternatives in an urban environment for selection of the first household within a cluster.

## Methods

### Setting

Maradi, the third largest city in Niger, is an administrative and commercial center. The official 2001 census population of Maradi was 165,000, although recent population estimates suggest the city is now almost double this size. Maradi is divided into 17 officially-recognized quartiers (neighborhoods), comprised of compounds within which one or several families live. A map of each quartier was available before the start of the survey.

The complete study in Maradi consisted of 30 clusters of 14 families each, although the alternative methods were tested in only two quartiers of the city due to time and human resource constraints. We selected one quartier in the center of the city and another on the periphery, both having approximately the same area. Oral informed consent was obtained from local authorities before performing the study.

Each method was conducted independently by different survey teams of two persons, each overseen by one supervisor, and with the same survey team conducting the same method in both quartiers. Before beginning the survey, teams were provided with training on both the method and the objective of the informal study. Survey teams were asked (1) to record the time taken to select the first household from arrival at a designated location on the border of the quartier; and (2) to describe any difficulties encountered in identifying the first household. For estimation of the time taken to select the first household, we considered both the advance preparation time (time before field implementation) and the time needed for survey teams in the field.

For illustrative purposes only, we also compared the vaccination coverage estimate obtained using the three methods [19]. Vaccination coverage estimates were obtained by interviewing the head of household on vaccination status of all members of the household. Further details on the complete survey methodology are available elsewhere (Grais & Guthmann, Epicentre internal report 2006, available from the authors on request). For all three methods, we used a sampling interval of every fifth compound instead of proximity sampling to minimize the cluster effect. One randomly-selected household within each compound was surveyed.

### Description of methods

#### Spin-the-pen method

We followed the spin-the-pen (random walk) method, which has been described in several manuals [4,5,20]. Little additional advance preparation time was required to conduct this method in the field once clusters were identified. The geographic center of the quartier was indicated by the supervisor and marked on the map. Survey teams went to this area, and selected a random direction by spinning a pen. As households in Maradi tended to be within walled compounds along roads, the number of compounds in the direction indicated by the pen was then counted to the edge of the quartier (defined by the map). One compound was then randomly selected, using a random number table, as the first to be included in the survey.

#### GPS method

**Figure 1:**
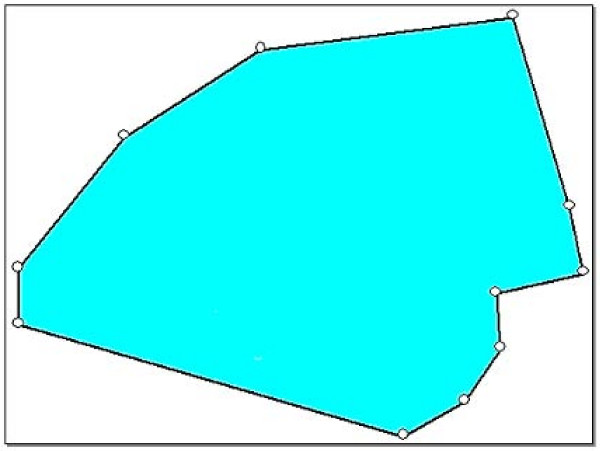
Sample map of one quartier in Maradi, Niger, drawn after GPS boundary points were defined.

Firstly, the external limits of the quartier, including its geographic coordinates, were delineated. This was accomplished with the aid of the "chef du quartier" who accompanied the survey team and supervisor in a car. A series of GPS points was taken for each change in direction along the perimeter of the quartier using a hand-held Garmin 12 XL GPS device (Olathe, KS, USA). According to the manufacturer's specifications, the handheld GPS is accurate to 15 meters. These coordinates were then entered into E-Pop software, [21] which both produces a map of the area and allows for random selection of the starting GPS point. Figure 1 shows an example of the quartier boundary drawn using GPS coordinates. The time taken to complete these steps was considered as the advance preparation time for this method. Once selected, the GPS point was re-entered into the handheld GPS as a waypoint and survey teams were then guided on foot to this location in the quartier to begin their survey. The closest compound to the right (with the teams facing north) of the GPS point was used as the first to be sampled.

#### Sampling grid method

**Figure 2:**
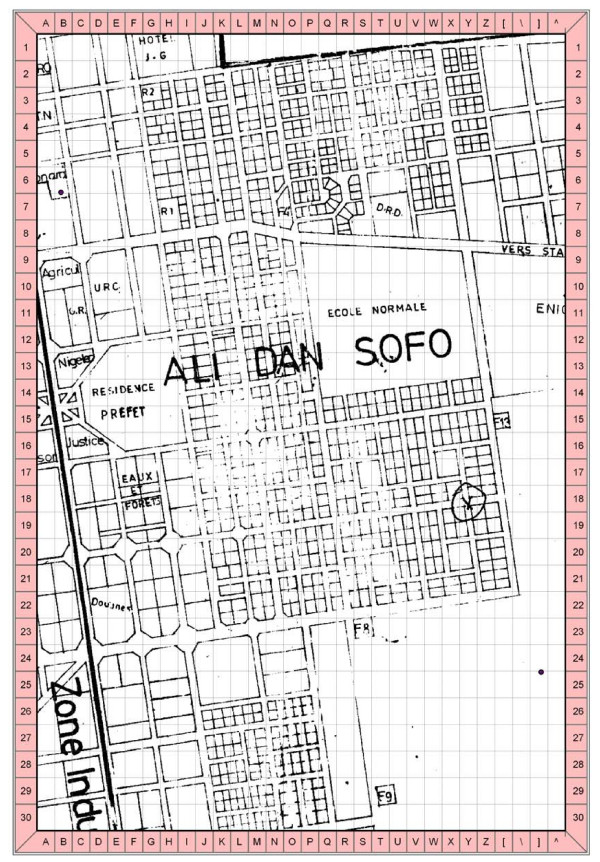
Example of a gridded map over one quartier in Maradi, Niger, as used by survey teams.

A sampling grid was superimposed onto a scanned street-map of the quartier using ArcGIS 9.1 (ESRI, Redlands, CA, USA). The sample grid consisted of *x *and *y *coordinates numbered from 1 to 30 (Figure 2). Using a random number table, a starting *x *and *y *coordinate on the grid were each selected. The time taken to complete these steps was considered as the advance preparation time for this method. Once in the field, teams located the selected *x*-*y *coordinate and the closest compound to the right (with the teams facing north) was used as the first to be sampled.

## Results

### Comparisons between the methods

The cluster survey was conducted during 26–29 April, 2006. Vaccine coverage estimates obtained were comparable using all three methods. These are provided in Table 1 for illustrative purposes only, to highlight the comparability of the methods rather than to provide meaningful estimates. The sampling grid method was accomplished in the least amount of time in both the central and peripheral quartiers, including the advance preparation time. Time spent when using the GPS method was comparable to the sampling grid method when advance preparation time was not included. The spin-the-pen method required the longest time, although little advance preparation was required. Table 2 shows the estimated time required for each method to be completed in each cluster.

**Table 1:** Vaccination coverage estimates using the three second-stage sampling methods

	**Central quartier**		**Peripheral quartier**	
	N*	**Vaccine coverage (95% CI)**	***N***	**Vaccine coverage (95% CI)**
	
**Spin-the-pen**	82	90.2 (81.2, 95.4)	76	75.0 (63.5, 83.9)
**GPS**	114	85.9 (77.9, 91.5)	84	77.4 (66.7, 85.5)
**Sample grid**	85	87.1 (77.6, 93.1)	84	78.6 (68.0, 86.5)

*Estimates include all household members.

**Table 2:** Time taken, using each of three different methods, for teams to select and find the first household in a cluster (expressed in minutes rounded to the nearest 5)

		**Central quartier**	**Peripheral quartier**
**Spin-the-pen**	Preparation	0	0
	Field	90	105
		
	**Total**	90	105
**GPS**	Preparation*	40	70
	Field	25	40
		
	**Total**	65	110
**Sample grid**	Preparation+	20	20
	Field	15	20
		
	**Total**	35	40

*Includes collection of GPS points along perimeter of quartier and selection of starting point.

+Includes scanning map of quartier, superimposing grid and selection of starting point.

According to the survey teams, the spin-the-pen method was the most difficult to implement, despite the fact that a map was available to identify the geographic center and the external limits of the quartier. Survey teams noted that if the pen indicated a direction close to a main road, this was chosen as the random direction. Survey teams spent the majority of their time counting compounds along the randomly-selected direction. A decision on which direction to take (i.e. interpretation of the direction indicated by the pen) was taken only after lengthy discussion. If a barrier was encountered along the transect, in order to continue, the barrier had to be bypassed; this often led to further lengthy discussion on how to proceed.

Although the sampling grid method presented the least technical or practical issues, it was not completely without difficulties. For example, verification of the intersection, which had been randomly chosen, was not easy. Streets were not well marked and required discussion to identify the starting location. The GPS method received the most enthusiastic feedback from survey teams, although this may be due to the use of new technology. The main difficulty encountered using the GPS was that navigation with the handheld device proved somewhat difficult in a dense urban environment due to physical barriers obstructing the route to the waypoint.

We summarize all the findings of the comparison of these methods in Table 3.

**Table 3:** Summary of comparison of the three different methods in an urban context, for the main criteria used

	**Time**	**Computer software/expertise needed**	**Cost**	**Advance preparation**
**Spin-the-pen**	+++	-	-	-
**GPS**	++	+++	++	++
**Sample grid**	+	+/- *	+/- *	+

*Depending on whether software is used vs a hand-drawn grid.

## Discussion

The results of this exploratory analysis suggest that the two alternative second-stage sampling methods investigated here can be performed quickly in an urban context, potentially yield similar results to the spin-the-pen random walk method, and may introduce less bias. The key advantage is that supervisory personnel can choose the starting point before fieldwork begins, reducing bias that might arise when starting points are chosen on the basis of convenience or when the choice relies heavily on survey team judgments. Both alternative methods were faster than spinning the pen and led to less debate over the choice of the first household. If geographic data of the survey site are available before the start of the survey, the GPS method could be accomplished even faster. In addition, although we superimposed the grid electronically, using costly software, this could also be accomplished in the field by hand, by simply drawing an evenly spaced grid on transparent film or paper and superimposing it on the map of the area to be surveyed.

These two alternatives address some of the problems inherent to spinning the pen, though they also have limitations and do not solve other issues discussed below. Firstly, we only examined their implementation in an urban environment. A map of the city was available by quartier, roads were navigable, and a vehicle was used to obtain geographic coordinates of the quartiers used in the study, both of which were of manageable size. In addition, we were not faced with the problem of multi-story dwellings. If this were the case, a large number of households would have been identified at the same location, necessitating an additional decisional algorithm for selecting the first household.

Although both alternative methods are promising in urban areas, their use in other contexts may not be as evident, particularly in areas where the population is not uniformly dense. The main drawback is that both methods favor the selection of households in lower density urban areas. If we envision a simple four-by-four sample grid superimposed on a population comprising both high and low density zones, one quadrat of the grid may contain 50% of the population, another 30%, and 10% each in the two remaining quadrats. In our case, and in many urban areas, the population is more uniformly distributed, lessening this potential. Ideally, the sample grid should be weighted by population size, although this is more time-consuming and requires additional information on population density that may not be readily available. GPS points should also optimally be selected not as random points, but as random households, but this again, necessitates the availability of more extensive data on household location and population.

In a rural area, both identifying the boundaries of the cluster and tracing the perimeter can be very time-consuming. In our case, we conducted two other cluster surveys concurrently in the surrounding rural areas of Maradi. We did not choose to test the two alternatives in the rural areas, however, as we did not have readily available maps and households were extremely dispersed. Further, in a remote rural area, containing the sparse dwellings of a scattered population, selection of a random starting point by GPS or grid coordinates could result in an unidentifiable first household. This could be remedied by implementing a decision rule in the field, involving re-sampling of a grid or GPS location, or by ensuring that a local guide were available to identify the nearest household to the point selected. However, this may not be any less biased than using a local guide to identify the center of a cluster.

Finally, although we compared (and present in Table 1) the vaccination coverage estimates using each method for just two separate clusters each (one in a central urban and the other in a peripheral urban zone), of course in reality comparison of single-cluster estimates would be inappropriate. This is because estimates from a single cluster are both unrepresentative of the total sample population and too small to make any meaningful judgment of the estimate obtained.

The benefit of this research is simply the generation of additional hypotheses and the identification of directions for further studies, as we only compared two alternative methods in two quartiers of one urban area. As the spin-the-pen method is used largely because of its ease and feasibility in certain settings, it has become a frequently used method for initial household selection. However, there is no real "gold standard" available with which to compare the length of time taken and the ease of use for all three methods. Investigators, especially when conducting cluster surveys in difficult settings, need to consider all available methods in order to select the most appropriate one for their survey needs. We were limited in this context to a cluster survey and were interested in examining simple changes to improve our existing protocol. There may be other alternative methods for selection of the first household, which are even faster or easier and even lower potential for bias than those tested here. Further research is still needed in this area and we encourage all survey investigators to test other methods where feasible, so that we can build on our knowledge base and improve on methods for future surveys. Future studies that fully test and/or compare each of the alternative methods would be welcome. Ideally, such research should comprise a complete cluster survey using each method in an urban setting. This would allow appropriate comparison of the estimate of the variable being measured, as well as cost and feasibility of each method used.

## Competing interests

The author(s) declare that they have no competing interests.

## Authors' contributions

RFG conceived the study and its implementation and drafted the manuscript. AMCR participated in the study coordination and revised the manuscript critically for important intellectual content. JPG participated in the implementation and coordination of the entire study and revised the manuscript critically for important intellectual content. All authors read and approved the final manuscript.
